# An immune response in the bumblebee, *Bombus terrestris *leads to increased food consumption

**DOI:** 10.1186/1472-6793-6-6

**Published:** 2006-07-17

**Authors:** Elizabeth R Tyler, Sally Adams, Eamonn B Mallon

**Affiliations:** 1 Department of Biology, University of Leicester, University Road, LE1 7RH, Leicester, UK

## Abstract

**Background:**

The concept of a costly immune system that must be traded off against other important physiological systems is fundamental to the burgeoning field of ecological immunity. Bumblebees have become one of the central models in this field. Although previous work has demonstrated costs of immunity in numerous life history traits, estimates of the more direct costs of bumblebee immunity have yet to be made.

**Results:**

Here we show a 7.5% increase in energy consumption in response to non-pathogenic immune stimulation.

**Conclusion:**

This increase in energy consumption along with other results suggests that immunity is one of the most important physiological systems, with other systems being sacrificed for its continuing efficiency. This increased consumption and maintained activity contrasts with the sickness-induced anorexia and reduced activity found in vertebrates.

## Background

The idea that the immune system is costly and must be traded off against other important physiological systems is a corner stone of the rapidly developing field of ecological immunity [1]. For example, only a costly immune system can provide an explanation for the many observed parasitism-immunity- reproduction patterns seen in nature [2].

This cost of immunity can be divided into two parts. First, there is the cost of having the immune system (including the cost of its evolution) and second, there is a separate cost of using it. It is this second cost that is most studied, largely due to the difficulty of partitioning the maintenance cost from other physiological systems. Most studies into the cost of immunity have looked at the tradeoffs that an immune response requires [1]. Fewer studies have addressed the cost of the immune response in other more direct currencies such as increased metabolic rate or food consumption [3]. To separate this immune cost from the cost of disease, requires the use of a non-pathogenic means of stimulating the immune response.

In vertebrate studies, immune responses require both energy and protein. Infection leads to a heightened metabolic state to support the upregulation of the immune system. Severe infections can lead to a 55% increase in resting metabolic rate in humans [4]. Equally, many defense mechanisms require significant supplies of amino acids [5]. Sepsis in humans can lead to a loss of up to 20% in total body protein [6]. However, sick vertebrates often show a decreased appetite, part of the well-studied sickness behaviour [7]. In the present study we attempted to measure both the energy and protein consumption during a non-pathogenic immune stimulation in the bumblebee, *Bombus terrestris*. However we were unable to use the protein data (see methods).

The bumblebee has become a valuable model in ecological immunology [8]. The temporal dynamics of the bumblebee's immune response have previously been studied [9]. The immune response assay (zone of inhibition) increases rapidly from 2 h to 12 h after the insult with a peak at 48 h followed by a slow decrease. A measurable effect is still detectable 14 days after the initial insult. The immune response has been show to significantly reduce the survival of starved bees compared to controls [10]. Another cost of this immune response is an interaction with the nervous system, such that immune-challenged bees perform poorly in memory tests [11,12]. We have shown that protein levels mediate this memory reduction. The effect disappears if the bees can increase their protein intake. We have also found a trade-off between the specific arm (defense against a gut trypanosome) and the general arm (encapsulation response) of the bumblebee immune response [13].

In this study we examine the effect on nutrient intake of an immune response. Lipopolysaccharide, part of the gram-negative bacterial cell wall has been shown to stimulate the bumblebee immune system without any parasite being present [10]. We will stimulate the bees' immune response and record both their food consumption and activity patterns. If the immune response is costly we might expect to see an increase in food consumption. However the opposite result is also possible, as in the sickness induced anorexia found in vertebrates [14]. We measure activity as it could be decreased if traded off against the immune response.

## Results

**Figure 1:**
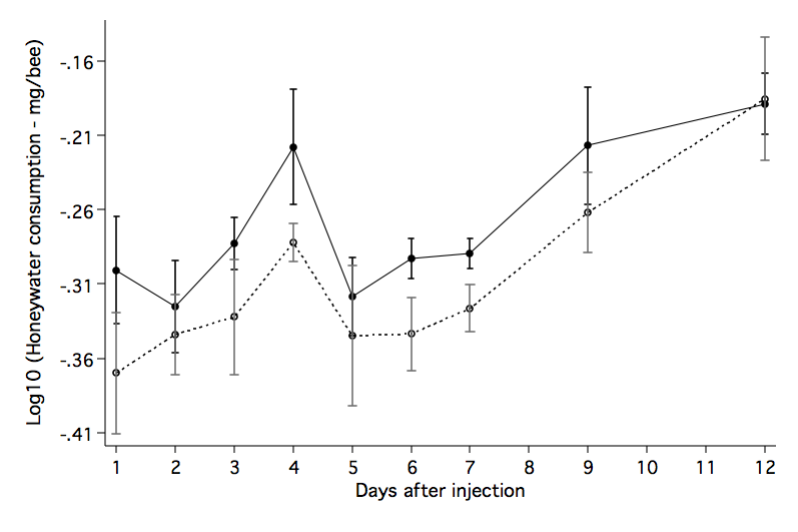
**Honeywater consumption over time**. Log_10 _transformed honeywater consumption per bee measured on 10 different days. The solid points represent the bees injected with LPS. The hollow points represent data from ringer injected bees. The t-bars represent the respective standard errors.

The rate of honeywater consumption was analysed using a repeated measures ANOVA. Log_10 _(honeywater consumption) was the dependent variable, colony and injection type were independent variables (between-individuals effect) and days after injection was a repeated measure (within-individuals effect). We detected no significant effect of colony origin on food consumption (F_1,5 _= 0.232, p = 0.650), consequently data for both colonies were combined for further analysis. Both day (F_8,56 _= 8.296, p, 0.0001) and injection type (F_1,7 _= 6.493, p = 0.038) alter food consumption. [See Figure 1].

We carried out an ANOVA with arcsine square root (proportion feeding) as the dependent variable and colony and injection type as independent variables with days after injection as a repeated measure. We detected no difference in observed feeding activity between injection types or days after injection, with on average 16.4 % (+/- standard deviation 11.6%) of the bees feeding at any given time (Treatment: F_1,6 _= 1.261, p = 0.304, day: f_4,24 _= 2.569, p = 0.064).

Colony origin had a significant effect on feeding activity with 10.6% (+/- 7.1%) of colony A bees feeding and 20.3% (+/- 12.4%) of colony B feeding (colony: F_1,6 _= 16.754, p = 0.006).

The pattern of movement was analysed with two separate contingency tables. There was no difference with injection type (χ^2^= 1.29, df = 2, p = 0.523) or day (χ^2 ^= 28.8, df = 18, p = 0.0516).

## Discussion

Immune stimulated bees consumed more honeywater than did control groups. There was no associated increase in the activity levels of immune stimulated bees. Honeywater consumption changed systematically over time. The consumption spike around day 4 coincides with the previously found maxima of immune response stimulated by both Ringer and LPS injections [9]. However the honeywater consumption continues to increase after this. We noticed that after day 7 we started to see male eggs produced. Worker bumblebees are not sterile. If the queen is removed they will begin to lay these male eggs [15]. The added energy requirement of egg production may explain the continued increase in energy consumption after day 6. This egg production cost would increase the variation in the days after injection factor, but would have no effect on our injection type result. We found no significant difference between injection types (LPS vs Ringer) in feeding activity. This combined with the honeywater consumption data suggests that the bees are eating more per feeding bout. Due to our inability to collect pollen data, we cannot say whether or how the immune challenge affected pollen consumption.

In a previous study severe food restriction did not affect the ability of a bumblebee to encapsulate a foreign particle [16]. Encapsulation, another part of the insect immune response, involves the prophenoloxidase system. Our study used LPS stimulation, which would lead to the production of antimicrobial peptides produced by the Imd pathway [17]. This does not explain the difference between our result and Schmid-Hempel's, as recently Freitak *et al*. found an increase in basal metabolic rate in a butterfly pupa due to encapsulation of a foreign particle [18]. If another costly activity is forced on the bumblebee, e.g. foraging, encapsulation was reduced [19]. These results taken together suggest, that immunity for insects is high in the hierarchy of important physiological systems, that is when an immune response is required the bee will eat more (our data), when food is restricted, energy from other systems will be diverted to keep the immune response at full strength [16]; and only if other activities are enforced on the insect [19] will immunity be compromised. This hypothesis could be tested by repeating Schmid-Hempel & Schmid-Hempel's experiment but with additional measurements of other physiological attributes (e.g. activity level). If the bee is diverting energy from other systems to keep immunity at full strength, we would predict its activity level to be reduced under food strain.

## Conclusion

Here we find an increase in the honeywater consumption of immune stimulated bees. There is no significant change in activity in the bees. This contrasts with the well-known anorexia and reduced activity which is part of the so called sickness behaviour of vertebrates. This response is also generated by LPS in vertebrates [14]. The vertebrate sickness behaviour is coordinated by cytokines. Almost nothing is known about insect cytokine biology [20]. It is possible that differences between vertebrate and insect physiology may explain these contrasting results. Recently, we have found that immune stimulated bees have decreased memory formation/recall abilities [11,12]. This mirrors a phenomenon found in vertebrates that is known to be modulated by cytokines [21]. Future work in our lab will attempt to understand these differences and similarities in insect and vertebrate immune modulated behaviours.

## Methods

### Injections

All bees used were between 5 and 7 days old when first injected. We challenged the bee's immune system by injecting, into the haemolymph, a dose of 5 μl of Ringer solution, containing 4% lipopolysaccharide (LPS, Sigma L-2755) (0.5 mg/ml = 9 mg 4% LPS g^-1 ^of bee). An experimental group of 25 bees were injected with LPS. To control for injection, another set of bees (n = 25) were injected with 5 μl of Ringer solution, a saline solution regularly used in insect physiology. The bees originated from two different colonies and were assigned to treatments such as to balance colony origin across treatments. After injection, bees were housed in colony/treatment groups (5 bees) for 13 days in plastic containers (17 × 13.5 × 9.5 cm). All bees were kept in constant red light at 26°C and 60% humidity.

### Energy calculation

Each bee box was daily provided with 1 g of pollen that had been left at 26°C 60% humidity for 24 hours to thaw and equilibrate. Pollen taken straight from the -20°C freezer gained weight for up to 14 hours (data not shown), presumably by absorbing water. Bees could also feed from 15 microtube caps that were filled with honey water (approximately 16 g). This amount ensured that the bees could feed *ad libitum*. An identically prepared container, with no bees, acted as a control for evaporation. We calculated a daily consumption per bee for both honeywater and pollen. Unfortunately, we were unable to use the pollen data as it was found that the bees added propolis to the pollen dish that interfered unpredictability with the pollen consumption estimates. These data were collected on days 1,2,3,4,5,6,7,9 & 12 of the experiment.

### Behavioural observations

The bees' behaviour was recorded for up to 13 days post injection. Behaviour was recorded at the same time each day (11.00 am). Each box was scanned 5 times with 5 minutes between scans. On each scan, each bee was classified as (1) stationary, (2) moving on the spot, or (3) moving. This data was collected on all 13 days of the experiment. We also recorded whether or not the bee was actually feeding. These data were collected on day 1,2,3,7,8.

## Authors' contributions

ERT carried out most of the experiments. SA carried out the remainder of the experiments and performed most of the analysis. EBM devised the initial experimental design and was largely responsible for the first draft of the manuscript.
